# Neurotization of free gracilis transfer with the brachialis branch of the musculocutaneous nerve to restore finger and thumb flexion in lower trunk brachial plexus injury: an anatomical study and case report

**DOI:** 10.6061/clinics/2016(04)03

**Published:** 2016-04

**Authors:** Yi Yang, Xue-jun Zou, Guo Fu, Ben-Gang Qin, Jian-Tao Yang, Xiang-Ming Li, Yi Hou, Jian Qi, Ping Li, Xiao-Lin Liu, Li-Qiang Gu

**Affiliations:** I The First Affiliated Hospital of Sun Yat-sen University, Department of Microsurgery and Orthopedic Trauma, Guangzhou, China; II Naval-Hospital, Department of Orthopedic Trauma, Guangzhou, China; III The First Affiliated Hospital of Henan University of Science and Technology, Department of Orthopedic Surgery, Luoyang, China

**Keywords:** Inferior Trunk of the Brachial Plexus Injury, Brachialis Muscle Branch of the Musculocutaneous Nerve, Gracilis, Muscle Transfer

## Abstract

**OBJECTIVE:**

To investigate the feasibility of using free gracilis muscle transfer along with the brachialis muscle branch of the musculocutaneous nerve to restore finger and thumb flexion in lower trunk brachial plexus injury according to an anatomical study and a case report.

**METHODS:**

Thirty formalin-fixed upper extremities from 15 adult cadavers were used in this study. The distance from the point at which the brachialis muscle branch of the musculocutaneous nerve originates to the midpoint of the humeral condylar was measured, as well as the length, diameter, course and branch type of the brachialis muscle branch of the musculocutaneous nerve. An 18-year-old male who sustained an injury to the left brachial plexus underwent free gracilis transfer using the brachialis muscle branch of the musculocutaneous nerve as the donor nerve to restore finger and thumb flexion. Elbow flexion power and hand grip strength were recorded according to British Medical Research Council standards. Postoperative measures of the total active motion of the fingers were obtained monthly.

**RESULTS:**

The mean length and diameter of the brachialis muscle branch of the musculocutaneous nerve were 52.66±6.45 and 1.39±0.09 mm, respectively, and three branching types were observed. For the patient, the first gracilis contraction occurred during the 4th month. A noticeable improvement was observed in digit flexion one year later; the muscle power was M4, and the total active motion of the fingers was 209°.

**CONCLUSIONS:**

Repairing injury to the lower trunk of the brachial plexus by transferring the brachialis muscle branch of the musculocutaneous nerve to the anterior branch of the obturator nerve using a tension-free direct suture is technically feasible, and the clinical outcome was satisfactory in a single surgical patient.

## INTRODUCTION

Traumatic brachial plexus injuries (BPI) are among the most severe peripheral nerve injuries in clinical practice and can cause permanent disability of the upper extremities 1. Several treatments are available for repairing upper trunk or total BPIs 2,3, whereas few methods exist for repairing injuries to the lower trunk of the brachial plexus. Among these treatments, nerve transfer and tendon transfer are two common approaches 4,5,6-7. Free gracilis muscle transfer can be used to restore elbow and finger function after devastating BPIs 8,9,10-11. The contralateral C7 (CC7) nerve and the intercostal nerve are mainly used as donor nerves to restore hand function when transferring the gracilis muscle 12. However, the outcomes of using these donor nerves are controversial. Due to the limited availability of usable donor nerves and unsatisfactory results, restoration of sufficient hand function following injury to the inferior trunk of the brachial plexus is still challenging. Recently, one surgeon successfully reconstructed the function of digit flexion by transferring the brachialis muscle branch of the musculocutaneous nerve (BMBMCN) to the posterior part of the median nerve in patients with C8–T1 avulsions 13. These patients showed low donor site morbidity after transection of the BMBMCN.

Due to the insufficient recovery of finger flexion when nerve transfer or tendon transfer is used after injury to the lower trunk of the brachial plexus, we present a new, effective treatment applied in one clinical case. This treatment is based on the anatomical finding that transferring the BMBMCN as a donor nerve to the anterior branch of the obturator nerve (ABON) of the gracilis can be used to re-enable thumb and finger flexion.

## METHODS

### Anatomical Study

Thirty formalin-fixed upper extremities from 15 adult cadavers (12 male and 3 female; 15 left and 15 right) donated to Southern Medical University in China were used in this study. The average age of the cadavers was 65 years (range, 31–73 years), and the average height was 170 cm (range, 154–175 cm). None of the specimens showed evidence of gross pathology, previous surgical procedures, or traumatic injuries to the upper extremities. The specimens were placed in the supine position with the upper limb in the abducted position. All the specimens were dissected by the same trained surgeon.

**Figure 1:**
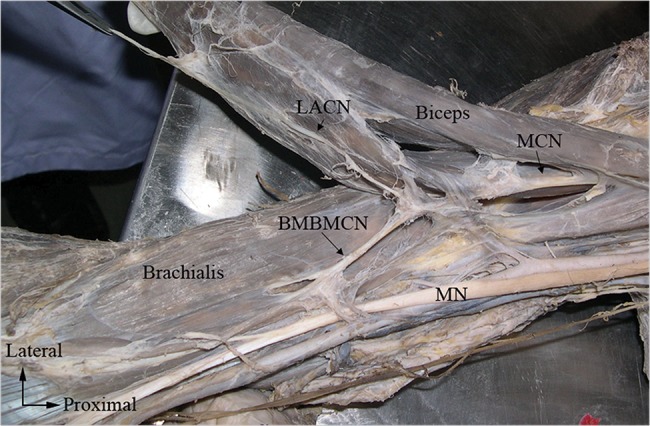
Photograph showing the nerve branching from the musculocutaneous nerve that innervates the brachialis muscle. Musculocutaneous nerve; Brachialis muscle branch of musculocutaneous nerve; Median nerve; Lateral antebrachial cutaneous nerve.

A longitudinal incision extending to the infraclavicular region was made to expose the musculocutaneous nerve and the BMBMCN at the middle and distal levels of the medial upper arm. The length and diameter of the BMBMCN and the musculocutaneous nerve were measured using Vernier calipers (Figure 1). The following additional measurements were obtained: 1 the distance from the origin of the BMBMCN to the midpoint of the humeral condylar; 2 the length and diameter of the BMBMCN; and 3 the course and the branch types of the BMBMCN. All dissections and observations were performed under 10x magnification.

### Clinical study

An 18-year-old male sustained a left BPI in a motorcycle accident and was transferred to our hospital 1 week after the injury. A physical examination showed near normal shoulder and elbow function, but the Horner sign was positive. Three months later, the patient recovered extension and partial flexion of his wrist. However, five months after the injury, no significant recovery of hand function was observed.

**Figure 2:**
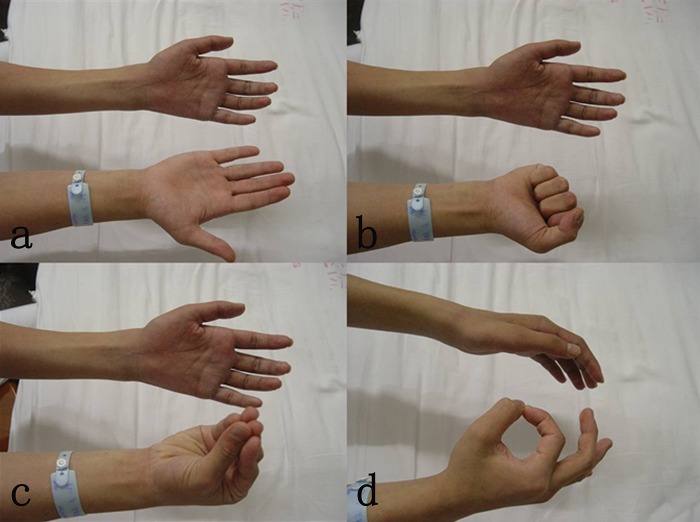
Photographs showing that the strength of finger and thumb flexion was M0.

The range of wrist flexion and extension was near normal but with radial deviation. Flexion, extension, opposition, abduction, and adduction of the digits were absent, and the involved muscles had a strength of M0 according to the British Medical Research Council (BMRC) grading system (Figure 2). The patient had decreased sensation in his radial three digits, and sensation was completely absent in the ring and little fingers. Magnetic resonance imaging (MRI) and electromyogram (EMG) testing indicated a C8 and T1 nerve root avulsion. Based on the results of the MRI evaluation, EMG testing and physical examination, the patient was diagnosed with a C8–T1 avulsion of the left brachial plexus.

### Surgical procedures

**Figure 3:**
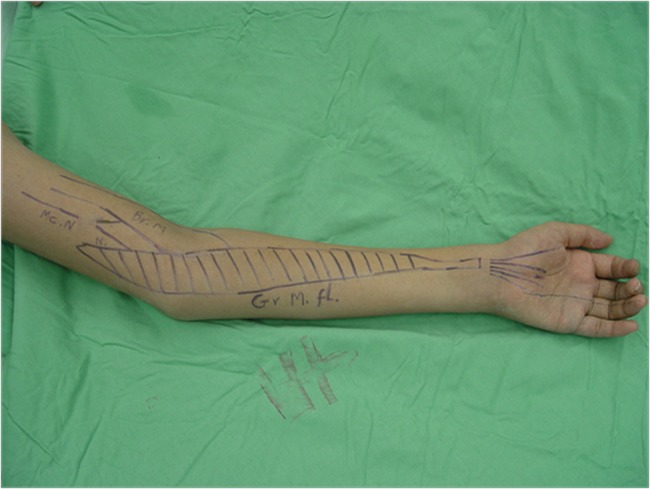
The preoperative surgical plan. Musculocutaneous nerve; Brachialis muscle; Gracilis muscle; flexion.

The surgical plan was discussed with the family and the patient before the operation (Figure 3). The operation was conducted under general anesthesia with the patient in the supine position. A 15-cm longitudinal incision was made along the medial and inferior surface of the medial upper extremity. The BMBMCN was located 14 cm proximal to the midpoint of the humeral condylar and was detached after local blockade with 1% lidocaine. The gracilis (approximately 46 cm in length) was harvested along with the ABON and the vascular supply from a branch of the profunda femoris artery and vein. A skin paddle (approximately 15×4 cm in length and width) was harvested to facilitate postoperative flap monitoring as well. The entire gracilis was taken and positioned in the recipient site.

The proximal part of the gracilis muscle was attached to the medial brachial intermuscular septum of the arm and sutured to the flexor digitorum profundus and flexor pollicis longus distally with double interlacing sutures. The gracilis artery originating from the profunda femoris artery was anastomosed to the brachial artery. Two veins of the gracilis pedicle were anastomosed to the basilica and deep brachial vein of the brachial artery in an end-to-end manner.

**Figure 4:**
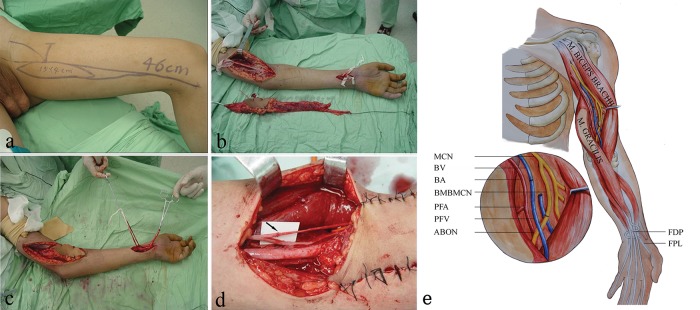
The gracilis was harvested along with the anterior branch of the obturator nerve and its vascular supply. A skin paddle was designed to facilitate postoperative flap monitoring (a). The harvested gracilis was placed beside the left arm (b). The harvested gracilis was placed inside the left forearm (c). The anterior branch of the obturator nerve of the gracilis was anastomosed to the brachialis muscle branch of the musculocutaneous nerve (arrow indicates the site of the anastomosis) (d). Schematic diagram of the operation (e).

The nerve of the gracilis was coapted to the BMBMCN using 9-0 nylon sutures with the assistance of a 10x surgical microscope (Figure 4).

The patient was required to wear a cast with 90° flexion of the elbow and fingers for 6 weeks after the surgery. He was advised to perform rehabilitation exercises, undergo electrical stimulation therapy, and take neurotrophic drugs. Elbow flexion power and hand grip were recorded according to BMRC standards, and the total active motion of the fingers was measured each month postoperatively. Our institutional review board (the First Affiliated Hospital of Sun Yat-sen University, China) approved this study, and informed consent was obtained from the participant.

### Statistical analysis

Data are presented as the means±SD. A paired t-test was used for continuous variables and to analyze differences between the left and right sides of the cadavers. Statistical significance was considered at a value of *p*<0.05. SPSS 13.0 (SPSS Inc., Chicago, IL, USA) was used to perform all statistical analyses.

## RESULTS

### Anatomical study

The microanatomical study of the 30 limbs of 15 adult cadavers showed that the brachialis muscle was innervated by the musculocutaneous nerve.

The mean lengths of the left and right sides of the BMBMCN were 52.60±6.62 and 52.72±6.50 mm, respectively. The overall mean length of the BMBMCN was 52.66±6.45 mm (range, 41.16–62.08 mm). The mean diameters of the left and right sides of the BMBMCN were 1.38±0.09 and 1.39±0.10 mm, respectively. The overall mean diameter of the BMBMCN was 1.39±0.09 mm (range, 1.20–1.56 mm). Based on the midpoint of the humeral condylar, the level of the BMBMCN origin ranged from 110.0 mm to 160.0 mm with a mean of 139.4±15.1 mm. The mean lengths were 139.3±15.3 and 139.5±15.4 mm for the left and right side, respectively (Table 1). No significant differences were observed between the sides.

Three branching types were observed in this anatomical study. Type I included a single branch and was present in 25 limbs (83.33%), whereas type II included two branches and was present in 1 limb (3.33%). Type III included multiple branches and was present in 4 limbs (13.33%) (Table 2).

### Clinical study

**Figure 5:**
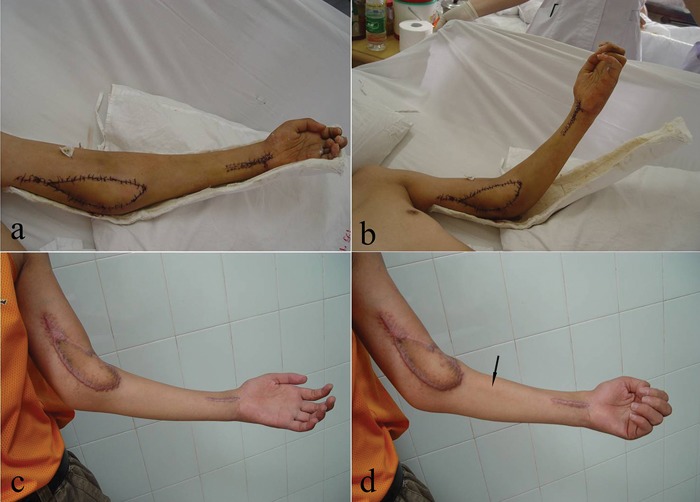
Photographs showing that the transection of the brachialis muscle branch of the musculocutaneous nerve did not cause functional impairment of the elbow when the cast was removed after the first month (a, b). One year after the operation, a noticeable improvement was observed in digit flexion due to gracilis contraction (arrow). The muscle power was M4 (c, d).

The patient's cast was removed after the first month. Four months after the surgery, an initial sign of gracilis contraction was observed. The muscle power was M3 at the 6th month. One year after the operation, a noticeable improvement (the muscle power was M4) was observed in digit flexion. The total active motion of the finger joints was excellent (209°). Transection of the BMBMCN did not cause functional impairment to the elbow or the wrist. The sensation levels in the radial 3 digits and the elbow flexion power involving the biceps, flexor carpi radialis (FCR), extensor carpi radialis longus (ECRL), and extensor carpi radialis brevis (ECRB) were similar to the preoperative levels (Figure 5).

## DISCUSSION

Motorcycle accidents are the leading cause of BPI, accounting for 54% of all BPI cases 14. Several different classification systems have been used for grading the severity of BPIs 15,16. BPIs are generally categorized as upper (C5-C7), lower (C8-T1) or total BPI (C5-T1). The incidence rates of C5-C7 and C5-T1 injuries are 42% and 48%, respectively, whereas the incidence of C8-T1 injuries is less frequent, with a rate of 3% 7.

### Rationale for the transfer of the BMBMCN for finger flexion reconstruction

The musculocutaneous nerve innervates the two elbow flexors, the biceps and the brachialis. The biceps covers the anteromedial 2/3 of the brachialis, whereas the brachioradialis covers the anterolateral 1/3 of the brachialis.

The brachialis is innervated by the musculocutaneous nerve as well as the radial or median nerve, or both of these nerves. Therefore, the brachialis potentially receives double or triple innervation 17. This multiple innervation may explain the preservation of normal elbow flexion function after BMBMCN transection.

### Anatomical basis for the surgical design of transfer of the BMBMCN for finger flexion reconstruction

One concern for transferring the BMBMCN to the ABON of the gracilis is the available length of the donor and recipient nerves. In the present study, the anatomical examination showed that the major branching pattern of the BMBMCN was type I single branching (83.33%). The mean length and diameter of the BMBMCN were 52.66±6.45 mm and 1.39±0.09 mm, respectively. These results are similar to those of other studies that have reported a mean length and diameter of the ABON of 8.7±2.1 cm and 2-2.6 mm 18,19, respectively. In this anatomical study, the minimum length of the BMBMCN was 41.16 mm. In addition, the gracilis tendon is of sufficient length, and the recipient area is abundantly supplied with arteries and veins; therefore, the position of the harvested gracilis can be adjusted to ensure a tension-free nerve anastomosis, even if the BMBMCN is relatively short in a patient due to anatomical variation. Ideally, the diameter of the donor and recipient nerve should match precisely. The minimum and average diameters of the BMBMCN are 1.20 mm and 1.39 mm; the diameter of the ABON is 2-2.6 mm 18,19. Thus, the BMBMCN is at least 46-60% of the ABON diameter, whereas the cross-sectional area of the BMBMCN is at least 21-36% of the ABON. According to the literature, the cross-sectional area of the three intercostal nerves is 17%-27% of the musculocutaneous nerve 20,21. Tötösy et al. also found that normal muscle force could be achieved with a minimum of 30% of the original motor neuron pool 22. Therefore, the length and diameter of the BMBMCN is not a limiting factor for suturing the nerve.

Another concern was the axon count of the donor nerve. Bhandari et al. reported that the number of myelinated fibers in the spinal accessory nerve was 1,671 23. Another study reported that 2090±462 myelinated fibers were present in the BMBMCN 24. Because more myelinated fibers are present in the BMBMCN than in the spinal accessory nerve and the spinal accessory nerve was used as the main donor nerve to reinnervate the gracilis, the BMBMCN has the potential for powerful reinnervation of the gracilis.

The methods for repairing the inferior trunk after BPI mainly include nerve transfer and tendon transfer. Unfortunately, neither of these treatments can achieve an ideal recovery. Gu Y et al. transferred the BMBMCN to the posterior 1/4 to 1/3 of the median nerve to restore finger flexion 13. However, this can jeopardize other fascicles of the median nerve, and this technique should be limited to early injury cases. Using the BMBMCN as a donor for gracilis transfer to restore finger and thumb flexion could avoid such unnecessary injury to the median nerve and reduce the potential for axon misrouting. Yang J et al. transferred the pronator teres branch to innervate the anterior interosseous nerve in a patient with a C8-T1 avulsion, which fully restored the range of motion of the fingers 5. The patient's finger flexor muscles regained grade 4 power; however, this method would not be feasible if only one branch was innervating the pronator teres. In another study, Bertelli et al. reported that transferring the supinator motor nerve to the posterior interosseous nerve effectively restored thumb and finger extension in patients with lower BPIs, but the outcome showed limited improvement. They also reported that transferring the brachialis muscle to the flexor digitorum profundus and flexor pollicis longus restored finger and thumb flexion 4. All of the patients described in that report achieved partial finger flexion, but the outcomes were not satisfactory. Goubier et al. transferred the ECRL to the flexor digitorum profundus and the brachioradialis tendon to the flexor pollicis longus tendon to restore thumb flexion in patients with lower BPI; all patients regained finger flexion 7. After the ECRL tendon transfer, wrist extension strength was decreased in some cases, but the ECRB muscle remained functional. In addition, initial recovery of shoulder and elbow movements may lead to a false expectancy for the recovery of hand function, which may result in late referral for treatment. Consequently, muscle atrophy will restrict the outcome of tendon transfer and nerve reconstruction. In this case, free muscle transfer might be the only choice for reconstructing hand function.

The BMBMCN has been used previously by different surgeons to repair C8–T1 brachial plexus avulsions and re-establish hand function 4,13,25,26. In this study, we used the BMBMCN as a donor nerve to reinnervate the gracilis, and the patient gained noticeable improvements in digit flexion and muscle power, which was graded as M4 one year after the operation. Transection of the BMBMCN did not cause functional impairment of the elbow or the wrist, which makes this technique useful for repairing injuries to the inferior trunk of the brachial plexus. Compared to nerve transfer or tendon transfer, a gracilis transfer can increase the number of functional muscles in the forearm without sacrificing the remaining hand and elbow functions in lower trunk BPI. In addition, from the time the patient was injured until he underwent the surgery, the muscles underwent a period of denervation and atrophy. Using the newly harvested gracilis could overcome such periods of muscle atrophy and provide better outcomes. This technique expanded the range and shortened the regeneration distance of the donor nerve in free muscle transfer. This procedure can be used as an initial treatment rather than a remedial treatment after primary nerve or tendon transfer fails. In addition, this method can be used in patients with late hospital referrals.

However, this technique should be restricted to inferior trunk BPIs because the musculocutaneous nerve originates at C5-C7. After middle trunk injury, the donor BMBMCN is likely to be affected, and the outcome of free gracilis transfer may not be satisfactory.

This study also had some limitations. First, we performed this technique on only one patient; more cases are needed for future tests of its clinical application. Second, although the patient showed satisfactory recovery of function after the relatively short follow-up period, long-term follow-up is needed to estimate the final outcome. Third, we did not perform a histomorphometric study to measure the number of myelinated fibers in the BMBMCN. In addition, muscle strength less than M3 cannot be measured by quantitative muscle power assessment and therefore is not conducive to continuous observation of the recovery of the transplanted muscle. As a result, the study used the BMRC for outcome assessment. Other objective measurements (including using a dynamometer to measure muscle strength, quantitative sensory testing, etc.) described in previous reports (27,28) should be used to assess the safety and effectiveness of this technique in the future. Finally, because this technique is technically demanding, it should be applied only for strict indications.

Based on this anatomical study, transferring the BMBMCN directly to the ABON with a tension-free suture is technically feasible. This surgical procedure successfully achieved adequate recovery of muscle power in the reinnervated gracilis and restoration of digit flexion without compromising elbow and wrist flexion following injury to the inferior trunk of the brachial plexus in a single surgical patient.

## AUTHOR CONTRIBUTIONS

Yang Y and Zou X were responsible for conception and design, acquisition of data and drafting the manuscript. Fu G and Qin BG revised the manuscript. Yang JT, Li XM and Hou Y contributed to analysis and interpretation of data. Qi J, Li P and Liu XL acquired data. Gu LQ was responsible for conception and design and acquisition of data. We declare that all the listed authors have participated actively in this study and meet the requirements of the authorship.

## Figures and Tables

**Figure 6:**
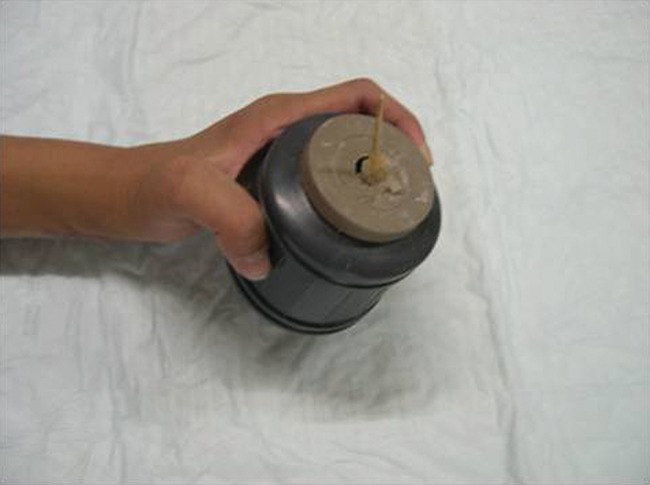
Photograph showing the patient holding a bottle at 12 months postoperatively.

**Table 1:** The measurements of the brachialis muscle branch of the musculocutaneous nerve (mean±SD, mm).

Items	Left (n=15)	Right (n=15)	Total (n=30)	t-value	*p*-value
Length	52.60±6.62	52.72±6.50	52.66±6.45	0.52	0.609
Diameter	1.38±0.09	1.39±0.10	1.39±0.09	0.397	0.698
Distance*	139.3±15.3	139.5±15.4	139.4±15.1	1.226	0.242

* Distance of the brachialis muscle branch of the musculocutaneous nerve origin to the midpoint of the humeral condylar.

**Table 2:** Branching type of the brachialis muscle branch of the musculocutaneous nerve.

Types	Left (n=15)	Right (n=15)	Total (n=30)
Type I, single branch	14	11	25(83.33%)
Type II, double branches	0	1	1(3.33%)
Type III, multiple branches	1	3	4(13.33%)

BMBMCN = brachialis muscle branch of the musculocutaneous nerve.
